# Bottom-Up Kinetic Chain in Drop Landing among University Athletes with Normal Dynamic Knee Valgus

**DOI:** 10.3390/ijerph17124418

**Published:** 2020-06-19

**Authors:** Nazatul Izzati Jamaludin, Farhah Nadhirah Aiman Sahabuddin, Raja Khairul Mustaqim Raja Ahmad Najib, Muhamad Lutfi Hanif Shamshul Bahari, Shazlin Shaharudin

**Affiliations:** 1 Exercise & Sports Science Programme, School of Health Sciences, Universiti Sains Malaysia, Kubang Kerian 16150, Kelantan, Malaysia; nazatulizzati096@gmail.com (N.I.J.); farhahsecret1996@gmail.com (F.N.A.S.); 130658.student.usm@gmail.com (R.K.M.R.A.N.); destructoidx18@gmail.com (M.L.H.S.B.); 2 Department of Mathematics and Science Education, Faculty of Education, University of Malaya, Kuala Lumpur 50603, Malaysia

**Keywords:** biomechanics, collegiate athletes, injury prevention, jump-landing

## Abstract

The study investigated the influence of ankle strength and its range of motion (ROM) on knee kinematics during drop landing. Fifteen male and fifteen female university athletes with a normal range of dynamic knee valgus (DKV) (knee frontal plane projection angle: men = 3° to 8°, females = 7° to 13°) were recruited. They performed drop landing at height 30 cm and 45 cm with three-dimensional motion capture and analysis. Knee angles were compared at specific landing phases. Isokinetic ankle strength was tested at 60°/s angular velocity while the weight-bearing lunge test was conducted to evaluate ankle ROM. For males, strength for both plantarflexors and dorsiflexors were associated with knee kinematics at both heights (30 cm: r = −0.50, *p* = 0.03; 45 cm: r = −0.45, *p* = 0.05) during maximum vertical ground reaction force (MVGRF) phase. For females, ankle invertor strength and knee kinematics were associated at both 30cm (r = 0.53; *p* = 0.02,) and 45 cm landing heights (r = 0.49, *p* = 0.03), while plantarflexor strength and knee kinematics showed a significant association during initial contact (r = 0.70, *p* < 0.01) and MVGRF (r = 0.55, *p* = 0.02) phases at height 30 cm only. Male and female athletes with normal range of DKV showed a significant relationship between ankle strength and knee kinematics at specific landing phases. These relationships varied with increased landing height.

## 1. Introduction

Dynamic knee valgus (DKV) is a mechanism of medial knee collapse due to a combination of hip internal rotation, hip adduction, knee valgus, and external rotation of the tibia during dynamic motions such as jump-landing [1]. Biomechanical factors observed from a poor technique of landing such as high impact loading, sudden decelerations, and high vertical ground reaction forces (GRFs) predispose athletes to lower limb injuries and pain [2]. Kinetic chain theory states that abnormalities of a joint may influence risks of injuries in other joints as observed in excessive DKV [3].

Fortunately, DKV is a modifiable factor of non-contact lower extremity injuries. Hence, previous studies conducted exercise intervention targeting the kinetic chain of DKV [4]. During closed chain activities, due to the interdependent of joint motions, excessive motions from one joint may overload subsequent tissues in the kinetic chain [5,6]. There are two types of kinetic chains related to DKV, which are top-down (i.e., proximal origins) and bottom-up (i.e., distal origins). Regarding the top-down kinetic chain, the function of muscles and other soft tissues either at the trunk or hip joint may influence the occurrence of altered kinematic patterns at the subsequent distal joints [7]. It was shown that weakness of hip musculature was associated with greater knee valgus during single leg ballistic and single leg squat tasks [6]. Hip and trunk muscle strengthening are commonly recommended to modify lower limb kinematics such as excessive hip medial rotation and adduction during weight-bearing tasks and to treat and prevent injuries at distal joints of lower limbs [8]. 

On the other hand, in the bottom-up kinetic chain, weakness of ankle musculature and foot structure may cause a lack of control at the knee joint and thus increase risks of knee injuries [9]. Khamis et al., [9] stated that DKV is often associated with the top-down kinetic chain of lower limbs. For instance, decreased isometric strength of hip abductors, adductors, and extensors was closely correlated with increased peak valgus angle at the knee [10]. Studies on the bottom-up kinetic chain of dynamic knee valgus are limited despite some evidence that pointed out the influence of the ankle joint on subsequent medial joints. For example, tibial rotation was significantly affected by ankle and foot kinematics [9]. Additionally, knee rotation was shown to be affected by toe directions (i.e., toe-in, toe-out, and natural position) [11]. However, the study by [11] was limited to physically active females who were not screened for excessive dynamic knee valgus.

Reduced dorsiflexion range of motion (ROM) is linked to increased knee valgus excursion during landing [12] and altered landing mechanics that predisposed athletes to injury [13]. Deficits in ankle dorsiflexion ROM may occur due to the decreased extensibility of the gastrocnemius/soleus complex and restricted posterior talar glide on the tibia, thus creating DKV [14]. A significant correlation was found between ankle dorsiflexion flexibility and the peak knee abduction angle (r = 0.355, *p* = 0.048) during landing [15]. Moreover, individuals with greater ankle dorsiflexion ROM demonstrated smaller GRFs and greater knee-flexion displacement during landing, which may be associated with a reduced risk of anterior cruciate ligament (ACL) injury [16]. 

In the present study, we investigated the association between knee kinematics during drop landing and ankle strength and its ROM among male and female university athletes. Previous studies by [17] and [18] did not exclude those with excessive DKV, which may influence their findings. It was shown that changes in ankle kinematics may cause excessive DKV or inward movement of the knee [19]. Furthermore, when strength was gender-matched among skilled athletes, the differences in hamstring and quadricep activity during landing were reduced despite biomechanical differences observed across gender [20,21]. Hence, when a biomechanically homogenous group is studied, the effects of landing heights could be less visible. Therefore, we aimed for a biomechanically homogenous group by including only those with normal DKV. The normal range of DKV, which can be assessed by the two-dimensional (2D) knee frontal plane projection angle (FPPA), is 3° to 8° for males and 7° to 13° for females [19]. We hypothesized that reduced ankle dorsiflexion ROM and ankle strength may be associated with knee angles during drop landing among university athletes with normal DKV. 

## 2. Materials and Methods

The protocol of this cross-sectional study was approved by the Human Research Ethical Committee of a local university (USM/JEPeM/18020138). A priori sample size calculation showed that 15 participants per gender were sufficient to yield 0.8 power of the study with 0.5 effect size [22]. The sample size calculation was conducted using GPower software (v.3.1.9.2, University of Düsseldorf, Düsseldorf, Germany). 

We recruited university athletes who participate regularly in sports at least three times per week and that had a normal body mass index (BMI: 18.5–24.9 kg/m^2^), aged between 19–25 years old, and exhibited a normal range of DKV during a drop vertical jump (DVJ). We included those with normal BMI to reduce the influence of body weight on knee biomechanics during landing. Those who were pregnant and had any lower limb injury at the time of data collection were excluded. After obtaining their information about medical history and written informed consent, anthropometric measurements such as their body weight, height, body fat percentage, and leg length were recorded. 

A screening test was conducted based on methods by [19] to distinguish those with and without excessive DKV. Briefly, markers were placed at the midpoint of the knee joint, midpoint of the center of the ankle joint, and anterior superior iliac spine (ASIS). Then, participants performed three trials of DVJ with one-minute rest interval between trials. The trials were captured from the frontal plane using a digital camera (SONY, Tokyo, Japan) and were further analyzed using Silicon Coach Pro v.8 (The Tarn Group, Dunedin, New Zealand). The two-dimensional (2D) knee FPPA is the intersection of the line created between the ASIS and center of the knee joint and the line between the center of the knee joint and the center of the ankle joint. The normal range of DKV is 7–13° for females and 3–8° for males [19]. The screening test took approximately 30 min to be completed. Upon analysis, participants were contacted regarding their results; those with excessive DKV were excluded from further tests. 

### 2.1. Drop Vertical Jump at Different Heights

**Figure 1:**
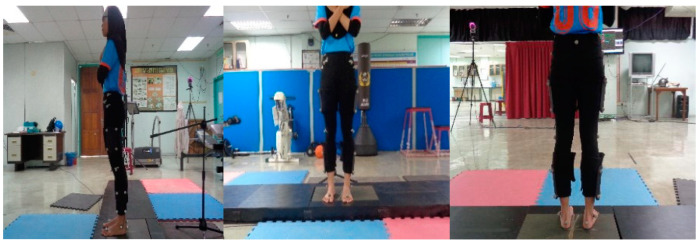
Plug-in-Gait marker placement.

Thirty-five reflective markers were attached bilaterally to the posterior superior iliac crest, anterior superior iliac spine, greater trochanter, medial and lateral knee, and medial and lateral malleolus based on the Plug-in-Gait marker set (Figure 1). 

Next, participants were required to drop off an adjustable plyometric box without an upward or forward jump action and adopted a stable double-leg landing posture to be considered a successful trial [18]. After a 5-minute rest interval, the test was repeated with different heights. The sequence of landing height (i.e., 30 and 45 cm) was randomized. Three successful trials at each landing height were selected for analysis. Upon completion, participants cooled down by cycling on a bike ergometer and stretching their legs. 

The trajectories of the reflective markers during these trials were sampled at 100 Hz and were identified using Qualisys Track Manager software (Qualisys, version 2.6.673, Gothenburg, Sweden). Then, the raw data of the marker coordinates were low-pass filtered using a fourth-order, zero-lag Butterworth filter with a cutoff frequency of 12 Hz by using Qualisys Track Manager software (Qualisys, version 2.6.673, Gothenburg, Sweden). The missing trajectories were pattern filled using spline estimates. Next, data were transferred to Visual 3D (version 5, C-Motion, Inc, Rockville, MD, USA) to construct a bone model and calculate the kinematic variables of the hip, knee, and ankle joint. 

### 2.2. Weight-Bearing Lunge Test 

**Figure 2:**
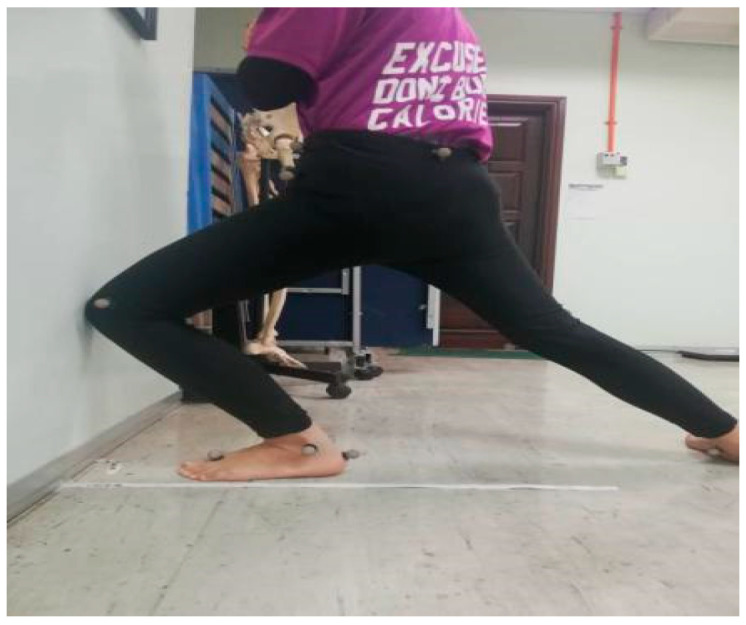
Position of the ankle range of motion test.

Maximum weight-bearing ankle dorsiflexion ROM was quantified in terms of maximum distance reached during the Weight-Bearing Lunge Test (WBLT). The test followed the procedure by Hoch et al., [17]. Briefly, the participants stood facing a wall with the tested foot at the front and parallel with a tape measure attached to the floor while the big toe touched the wall (Figure 2). The uninvolved foot was placed comfortably behind the tested foot. Next, participants lunged until their knee touched the wall while the heel remained firmly planted on the floor. This was to ensure that the foot posture did not influence the measurement. Then, they were asked to step backward in 1 cm increments until heel or knee contact could no longer be sustained during the lunge [17]. The maximum lunge distance was measured from the tip of the big toe to the wall in the nearest 0.1 cm. The WBLT was repeated for three trials for each leg, and the values were averaged for further analysis. 

### 2.3. Isokinetic Ankle Strength Test

After at least 24 hours of rest, participants performed isokinetic ankle strength tests using a dynamometer (Biodex System 3 Pro, Shirley, NY, USA). They were seated with 90° knee flexion and a neutral position of the ankle, while keeping their back straight on a chair with a Velcro strap attached to a strain gauge placed around the lower leg and foot. The ankle axis was set on the same line as the equipment axis, and the handles were held by both hands. We followed the Biodex manual, whereby the ankle axis was determined based on the head of the talus. Ankle strength in dorsiflexion/plantarflexion and eversion/inversion motions was tested in concentric mode at angular velocity of 60°/s for three sets of five repetitions per set and 120 s rest interval between sets. The data were averaged for further analysis. Ankle strength was measured in terms of peak torque per body weight (PT/BW, %). The antagonist:agonist ratio was determined by dividing the PT/BW of the antagonist muscle group by the PT/BW of the agonist muscle group. 

### 2.4. Statistical Analysis 

Data were tested for normal distribution with the Shapiro-Wilk test which is appropriate for small sample sizes (<50 samples) [22]. The kinematics data observed in the two different landing heights were compared across three different phases of landing, namely, initial contact (IC), maximum vertical ground reaction force (MVGRF), and maximum knee flexion (MKF) phases. IC was defined as the point in the trial when the vertical GRF exceeded 10 N [17]. The ankle dorsiflexion ROM and isokinetic ankle strength were compared between female and male collegiate athletes by using an independent T-test. Then, the relationships between ankle dorsiflexion ROM, ankle strength, and knee kinematic during landing at different heights were determined by using Pearson correlation coefficients. All statistical analyses were performed using the Statistical Package for the Social Sciences (SPSS) (version 22.0, IBM Corp., Armonk, NY, USA). The level of significance was set at *p* < 0.05.

## 3. Results

The two-dimensional knee FPPA during the DVJ screening test was significantly different across gender with *p*-value < 0.001 (male = 6.20° ± 1.55, female = 10.19° ± 1.91).

The physical characteristics of the participants were compared across gender. As expected, significant differences were observed in all physical characteristic variables across male (n = 15) and female (n = 15) university athletes (Height: male = 163.67 cm ± 7.19, female = 155.80 cm ± 4.11, *p*-value = 0.01; body fat percentage: male = 10.19% ± 5.15, female = 22.12% ± 3.02, *p*-value = 0.01). No significant differences across gender were observed for body weight (male = 55.56 kg ± 9.80, female = 56.77 kg ± 3.68, *p*-value = 0.66).

Weight-bearing ankle dorsiflexion ROM was not significantly different across gender (male = 33.45 cm ± 7.56, female = 36.83 cm ± 4.18, *p*-value = 0.14). 

No statistically significant differences in the isokinetic strength of the evertors, dorsiflexors, and antagonist:agonist ratios were observed across gender (Table 1). Males showed statistically greater strength in invertors and plantarflexors than females.

The knee angles during three specific landing phases from heights 30 cm and 45 cm are tabulated in Table 2. 

The relationships between knee kinematic and weight bearing ankle dorsiflexion (Table 3) and isokinetic ankle strength (Table 4) were presented according to gender.

## 4. Discussion

Among male athletes, significant relationships were observed between plantarflexor strength and knee kinematic at 30 cm landing height during maximum vGRF and maximum knee flexion (MKF) phases and at 45 cm landing height during the maximum vGRF phase. Also, a significant relationship was noted between dorsiflexor strength and knee kinematic at 30 cm landing height during maximum vGRF and MKF phases and at 45 cm landing height during the maximum vGRF phase only. Additionally, inverse relationships were observed between plantarflexor and dorsiflexor strength and knee kinematic during landing at both heights. These findings indicated that greater strength of the plantarflexor or dorsiflexor may cause the male athletes to land with a higher varus knee angle.

Regarding female athletes, a significant relationship was noted between the invertor strength and knee kinematic during the MKF phase for both landing heights. This proportional relationship indicates that a greater invertor strength may cause them to land in the valgus knee position. Decreased neuromuscular control of ankles may cause increased inversion angular velocity which is possibly a key contributor to recurrent ankle injuries [23]. In addition, a significant relationship was observed between plantarflexor strength and knee kinematics during 30 cm landing height at IC and maximum vGRF phases. The direct relationship shows that the greater the plantarflexor strength, the lower the tendency to land in the varus position. Indeed, it was previously shown that the knee was the primary shock absorber for both genders, whereas the ankle plantar-flexor muscles were the second largest contributor to energy absorption in females [24]. Moreover, knee kinematics were also significantly related to the evertor: invertor strength ratio at 30 cm landing height during the MKF phase only and at 45 cm landing height during all phases. An increased evertor:invertor strength ratio implies greater chances of landing in the varus position. Among those with chronic ankle instability (CAI) and coper athletes, ankle eversion was frequently used as their major adaptation strategy in the initial landing phase to reduce the effect of ankle over-inversion [25].

Knee kinematic and ankle strength showed an inverse relationship in males, but a proportional relationship in females. Males had greater vertical leg stiffness compared to females, which occurred with a lower center of mass (COM) vertical displacement per height and greater peak GRF per body weight [26]. A previous study showed that males had greater ankle joint stiffness, a lower initial plantar flexion angle, lower ankle ROM, and greater changes and peaks in ankle moment compared to females [26]. Ankle joint stiffness was thought to be due to a reduced ankle joint ROM during landing. 

Peak vertical and posterior GRF increased with greater vertical height at landing [27]. Increased height may elevate maximum vGRF experienced during landing, thus with increased risks of sustaining traumatic injuries such as ACL rupture [18]. Moreover, a previous study conducted among male athletes showed that maximum vGRF was negatively correlated with ankle plantarflexion at increasing vertical landing height [27], which is similar to our findings. However, studies that exclude females with excessive DKV are not known for comparison with our findings.

Ankle dorsiflexion ROM among female athletes showed a positive relationship with knee kinematic during all phases of landing at 30 cm height. Similarly, Brookreson [28] observed a positive linear relationship (r = 0.75, *p* = 0.001) between weight-bearing of dominant ankle dorsiflexion ROM and knee kinematics at 40 cm landing height during the MKF phase [28]. Greater passive ankle-dorsiflexion ROM was associated with greater knee-flexion displacement and smaller GRFs during landing, which may be associated with a reduced risk of ACL injury [29]. However, their study involved only thirteen physically active male athletes who were proficient in landing and jumping techniques and free from lower limb injury, and no screening test was conducted to exclude people with excessive DKV. Additionally, male athletes are likely to demonstrate increased stiffness in ankle, knee, and hip joints with maturation [27]. 

We noted that there were no statistically significant differences in ankle dorsiflexion ROM between male and female university athletes (Table 2). In a study among 107 of healthy university students, the average values for total distance covered was 32.7 cm for males and 33.9 cm for females [28]. Our findings on total distance covered are within the range of the findings by Hankemeier & Thrasher [30], who also conducted WBLT on the dominant leg only. 

In a previous study among 128 healthy adults (55 men, 73 women), ankle dorsiflexion ROM for females was greater than for males, although the differences were not statistically significant [28]. Additionally, females showed greater ROM in lower-extremity joints than adult males [31]. The results from these two studies concur with our findings. Greater ankle mobility among females is due to the greater capacity of plantarflexors, as compared to males [31]. Greater passive ankle dorsiflexion ROM was associated with greater hip and knee flexion and lower GRFs during a jump-landing task in healthy individuals [15]. Dorsiflexion deficits may limit the ability to fully achieve a closed-packed, stable position of the ankle during dynamic activities, such as gait and jump-landing [17]. Hence, athletes and coaches should focus on improving ankle ROM in their jump-landing training to prevent injury.

Isokinetic strength was normalized to body weight and expressed as a percentage to reduce the influence of participants’ body weight on results. We observed that males exerted greater peak torque/body weight (PT/BW) for ankle inversion than females (*p* = 0.01) (Table 1). Also, males showed higher plantarflexion PT/BW than females (*p* = 0.02). Males have greater muscle mass especially in lower extremities than females, which may help them to achieve higher PT/BW during the isokinetic plantarflexion test [31].

There are some limitations in the current study that should be addressed in future studies. Firstly, our participants were barefoot during trials, which may not represent the real condition of sporting activities. Bare feet are preferable because wearing shoes during trials may influence the athletes’ landing strategies due to the shoes’ shock absorption effect [2,32]. Secondly, although we ensured that the participants maintained their foot position during WBLT, the foot posture (i.e., pronation and supination) was not quantified. It was also shown that foot position did not influence knee kinematics during single leg squats among male athletes with normal DKV [33]. Future studies are recommended to investigate the relationship between foot structure (i.e., posture and arch) and landing mechanics. Finally, our results are limited to physically active young adults due to their greater exposure to injury risks than other members of the population. 

## 5. Conclusions

Male athletes with normal DKV showed a consistent relationship between knee FPPA and plantarflexor strength at both landing heights, particularly during the maximum vGRF phase. Moreover, dorsiflexor strength was significantly associated with knee FPPA at both landing heights during the maximum vGRF phase. In female athletes with normal DKV, invertor strength was associated with knee FPPA at 30 cm during the MKF phase, while plantarflexor strength and knee FPPA showed a significant association during IC and maximum vGRF at 30 cm landing height only. Knee FPPA was also significantly associated with evertor:invertor strength during all landing phases at 45 cm. Ankle ROM was significantly correlated with knee FPPA at 30 cm landing height only. Also, male and female athletes with normal DKV showed a significant relationship between ankle strength and knee FPPA at specific landing phases. These relationships varied when the jump height increased. 

## Figures and Tables

**Table 1:** Comparison of isokinetic ankle strength across male and female university athletes (*n* = 30).

Ankle Strength (Peak Torque/Body Weight)	Mean (SD)		*p*-Value
	Male (*n* = 15)	Female (*n* = 15)	
Eversion	113.40 (13.42)	119.95 (13.07)	0.77
Inversion	136.49 (21.51)	114.41 (17.49)	0.01 *
Strength ratio (Evertors: Invertors)	0.85 (0.18)	0.98 (0.21)	0.08
Plantarflexion	373.62 (24.44)	336.52 (24.44)	0.02 *
Dorsiflexion	150.35 (27.31)	140.45 (24.27)	0.30
Strength ratio (Plantarflexors: Dorsiflexors)	2.517 (0.30)	2.45 (0.35)	0.55

* indicates a statistically significant difference between gender. SD: standard deviation.

**Table 2:** Knee angle during phases of landing from 30 cm and 45 cm heights across male and female university athletes (*n* = 30).

Phases of Landing	Knee Angle (°)		*p*-Value
	Male (*n* = 15)	Female (*n* = 15)	
At 30 cm Initial Contact	1.56 (4.86)	−2.04 (4.09)	0.05 *
Maximum vGRF	1.85 (7.52)	−2.00 (5.19)	0.11
Maximum knee flexion	3.45 (8.82)	−1.01 (7.08)	0.14
At 45 cm Initial Contact	0.29 (4.20)	0.60 (6.31)	1.00
Maximum vGRF	−0.17 (6.45)	1.05 (7.09)	0.70
Maximum knee flexion	2.23 (8.20)	3.80 (6.93)	0.37

* indicates a statistically significant difference between gender; (−) indicates knee abduction; (+) indicates knee adduction. vGRF = vertical ground reaction force.

**Table 3:** Relationship between ankle dorsiflexion range of motion and knee angle during landing among male and female university athletes (*n* = 30).

Weight-Bearing Ankle Dorsiflexion		30 cm Landing Height			45 cm Landing Height		
	Value	IC	MV GRF	MKF	IC	MV GRF	MKF
Males (*n* = 15)							
Dorsiflexion range of motion (cm)	P	0.14	0.25	0.46	0.15	0.47	0.16
	R	−0.30	−0.19	0.03	−0.28	0.02	0.28
Females (*n* = 15)							
Dorsiflexion range of motion (cm)	P	0.05	0.04 *	0.05	0.35	0.20	0.14
	R	0.44	0.46	0.43	0.11	0.24	0.29

* indicates a statistically significant association. IC = initial contact, MVGRF = maximum vertical ground reaction force, MKF = maximum knee flexion.

**Table 4:** Relationship between ankle strength and knee frontal plane projection angle (FPPA) during landing in male and female university athletes (*n* = 30).

Isokinetic Ankle Strength	Value	30 cm Landing Height			45 cm Landing Height		
		IC	MV GRF	MKF	IC	MV GRF	MKF
Males (*n* = 15)							
Evertors	P	0.43	0.39	0.35	0.29	0.36	0.26
	R	−0.05	0.07	0.11	0.16	0.10	0.18
Invertors	P	0.48	0.27	0.25	0.32	0.28	0.42
	R	0.15	0.17	0.19	0.13	0.17	0.54
Plantarflexors	P	0.12	0.03 *	0.01 *	0.27	0.05 *	0.36
	R	−0.32	−0.50	−0.59	−0.18	−0.45	−0.48
Dorsiflexors	P	0.09	0.03 *	0.05 *	0.15	0.05 *	0.09
	R	−3.59	−0.50	−0.28	−0.28	−0.45	−0.36
Evertors: Invertors	P	0.44	0.37	0.42	0.50	0.38	0.42
	R	−0.04	−0.04	−0.06	−0.00	−0.08	0.06
Plantarflexors: Dorsiflexors	P	0.23	0.24	0.48	0.21	0.32	0.42
	R	0.21	0.20	−0.11	0.22	0.13	−0.05
Females (*n* = 15)							
Evertors	P	0.39	0.49	0.43	0.31	0.50	0.40
	R	−0.08	−0.01	−0.05	−0.14	0.01	0.07
Invertors	P	0.17	0.06	0.02 *	0.25	0.09	0.03 *
	R	0.27	0.43	0.53	0.19	0.36	0.50
Plantarflexors	P	0.00 *	0.02 *	0.10	0.22	0.18	0.41
	R	0.70	0.55	0.35	−0.22	−0.26	−0.07
Dorsiflexors	P	0.12	0.20	0.29	0.44	0.36	0.28
	R	−3.59	0.23	0.15	−0.04	−0.10	0.17
Evertors: Invertors	P	0.21	0.10	0.03 *	0.05 *	0.04 *	0.03 *
	R	−0.23	−0.35	−0.51	−0.44	−0.48	−0.51
Plantarflexors: Dorsiflexors	P	0.48	0.47	0.48	0.42	0.48	0.20
	R	−0.01	0.02	−0.01	−0.06	−0.01	−0.23

* indicates a statistically significant association. IC = initial contact, MVGRF = maximum vertical ground reaction force, MKF = maximum knee flexion.
